# Clathrin light chain‐conjugated drug delivery for cancer

**DOI:** 10.1002/btm2.10273

**Published:** 2022-11-28

**Authors:** Sungwook Jung, Liwei Jiang, Jing Zhao, Leonard D. Shultz, Dale L. Greiner, Munhyung Bae, Xiaofei Li, Farideh Ordikhani, Rui Kuai, John Joseph, Vivek Kasinath, David R. Elmaleh, Reza Abdi

**Affiliations:** ^1^ Transplantation Research Center, Renal Division Brigham and Women's Hospital, Harvard Medical School Boston Massachusetts USA; ^2^ Institute of Health and Medical Technology Hefei Institutes of Physical Science, Chinese Academy of Sciences Boston Hefei China; ^3^ Department of Immunology The Jackson Laboratory Bar Harbor Maine USA; ^4^ Department of Molecular Medicine University of Massachusetts Medical School Worcester Massachusetts USA; ^5^ Department of Biological Chemistry and Molecular Pharmacology Harvard Medical School Boston Massachusetts USA; ^6^ Center for Nanomedicine and Division of Engineering in Medicine, Department of Medicine Brigham and Women's Hospital, Harvard Medical School Boston Massachusetts USA; ^7^ Department of Radiology Massachusetts General Hospital, Harvard Medical School Boston Massachusetts USA

**Keywords:** cancer, drug delivery, fluorescence imaging, targeted therapy

## Abstract

Targeted drug delivery systems hold the remarkable potential to improve the therapeutic index of anticancer medications markedly. Here, we report a targeted delivery platform for cancer treatment using clathrin light chain (CLC)‐conjugated drugs. We conjugated CLC to paclitaxel (PTX) through a glutaric anhydride at high efficiency. Labeled CLCs localized to 4T1 tumors implanted in mice, and conjugation of PTX to CLC enhanced its delivery to these tumors. Treatment of three different mouse models of cancer—melanoma, breast cancer, and lung cancer—with CLC‐PTX resulted in significant growth inhibition of both the primary tumor and metastatic lesions, as compared to treatment with free PTX. CLC‐PTX treatment caused a marked increase in apoptosis of tumor cells and reduction of tumor angiogenesis. Our data suggested HSP70 as a binding partner for CLC. Our study demonstrates that CLC‐based drug‐conjugates constitute a novel drug delivery platform that can augment the effects of chemotherapeutics in treating a variety of cancers. Moreover, conjugation of therapeutics with CLC may be used as means by which drugs are delivered specifically to primary tumors and metastatic lesions, thereby prolonging the survival of cancer patients.

**Abbreviations**
ACN: acetonitrile
ADC: antibody‐drug conjugate
BUN: blood urea nitrogen
CD31: cluster of differentiation 31
CLC: clathrin light chain
DAPI: diamidino‐2‐phenylindole
DLS: dynamic light scattering
DMEM: Dulbecco's modified Eagle's medium
DMSO: dimethyl sulfoxide
ECM: extracellular matrix
EDC: 1‐ethyl‐3‐(3‐dimethylaminopropyl)carbodiimide
ESIMS: electron spay ionization mass spectrometry
*E. coli*
: *Escherichia col*i
FBS: fetal bovine serum
GAPDH: glyceraldehyde‐3‐phosphate dehydrogenase
HPLC: high‐performance liquid chromatography
HRP: horseradish peroxidase
HSP70: heat shock protein 70
H&E: hematoxylin and eosin
LLC1: Lewis lung carcinoma 1
LYVE1: lymphatic vessel endothelial hyaluronan receptor 1
MALDI‐TOF: matrix‐assisted laser desorption ionization time‐of‐flight
MFI: mean fluorescence intensity
MS: mass spectrometry
NHS: *N*‐hydroxysuccinimide
NMR: nuclear magnetic resonance
PDB: protein data bank
PK: pharmacokinetics
PTX: paclitaxel
ROI: region of interest
RPMI: Roswell park memorial institute
SD: standard deviation
SDS‐PAGE: sodium dodecyl sulfate‐polyacrylamide gel electrophoresis
siRNA: small interfering ribonucleic acid
TDLN: tumor‐draining lymph node
*TGI*
: tumor growth inhibition
UV–Vis: ultraviolet–visible

## 1 INTRODUCTION

Targeted drug delivery systems can amplify the concentration of transported payloads at various tissues of interest, including tumors.^1^, ^2^, ^3^ Thus, a key advantage of targeted drug delivery is its simultaneous enhancement of the therapeutic index of the drug, along with a reduction in its systemic exposure and overall toxicity.^3^

Antibody‐drug conjugates (ADCs) are emerging platforms for the delivery of a range of cancer drugs.^4^, ^5^ Monoclonal antibodies have attracted major interest as vehicles for the delivery of cancer therapeutics by recognizing specific target antigens overexpressed on the surface of cancer cells. The molecular weight of antibodies affects the tumor penetration of ADCs, and the large size of IgG antibodies (~150 kDa) used widely for current ADCs presents a notable challenge.^6^ The immunogenicity of antibodies also results in systematic toxicity and their rapid clearance from the body, culminating in the delivery of a small fraction of the administered drug.^7^ Therefore, efforts in identifying alternative approaches to achieve targeted therapy have heightened. One such route is the use of a small protein for disease treatment.^8^, ^9^, ^10^ Advantages to the use of small proteins include easy and affordable production, high pharmaceutical potency and flexibility, as well as low toxicity in sequence and conjugation possibilities.

Here, we report a novel application of clathrin light chain A (CLC)‐based drug delivery. CLC is an endogenous small protein that forms a network of triskelions that constitute a polyhedral lattice around vesicles that assist in the sorting of cargo for intracellular trafficking.^11^ Clathrin‐coated vesicles play an essential role in cellular membrane trafficking. The clathrin triskelion consists of three heavy chains and three light chains.^11^ During clathrin‐mediated endocytosis, this cytosolic clathrin triskelion interacts with other cytosolic proteins, including HSP70, and regulates the formation of clahtrin‐coated vesicles.^12^ The light chain is a primary functional unit of this triskelion via its interaction with calcium ions^13^ or calmodulin,^14^ or through its phosphorylation.^15^ Endogenous proteins, such as ferritin and albumin, have attracted great interest in the field of drug delivery, due to their biocompatibility and favorable safety profiles.^16^, ^17^, ^18^, ^19^, ^20^, ^21^, ^22^ In the same vein, CLC piqued our interest as a novel endogenous small protein that could be used as a carrier of payloads.

First, we confirmed that CLCs interact with cancer cells and could be used as vehicles for targeted delivery to malignant tumors. Next, CLCs were conjugated to the antineoplastic agent paclitaxel (PTX) via reaction with glutaric anhydride (CLC‐PTX) to evaluate their utility for cancer‐targeted delivery and treatment in a series of mouse models. CLC‐PTX enhanced the concentration of PTX at the tumor site and suppressed tumor growth in mouse models of breast cancer, melanoma, and lung carcinoma. In addition, CLC‐PTX was found to reduce the size of metastatic lesions in breast cancer.

## 2 RESULTS

### 2.1 We synthesized and characterized CLC‐PTX conjugates

**FIGURE 1:**
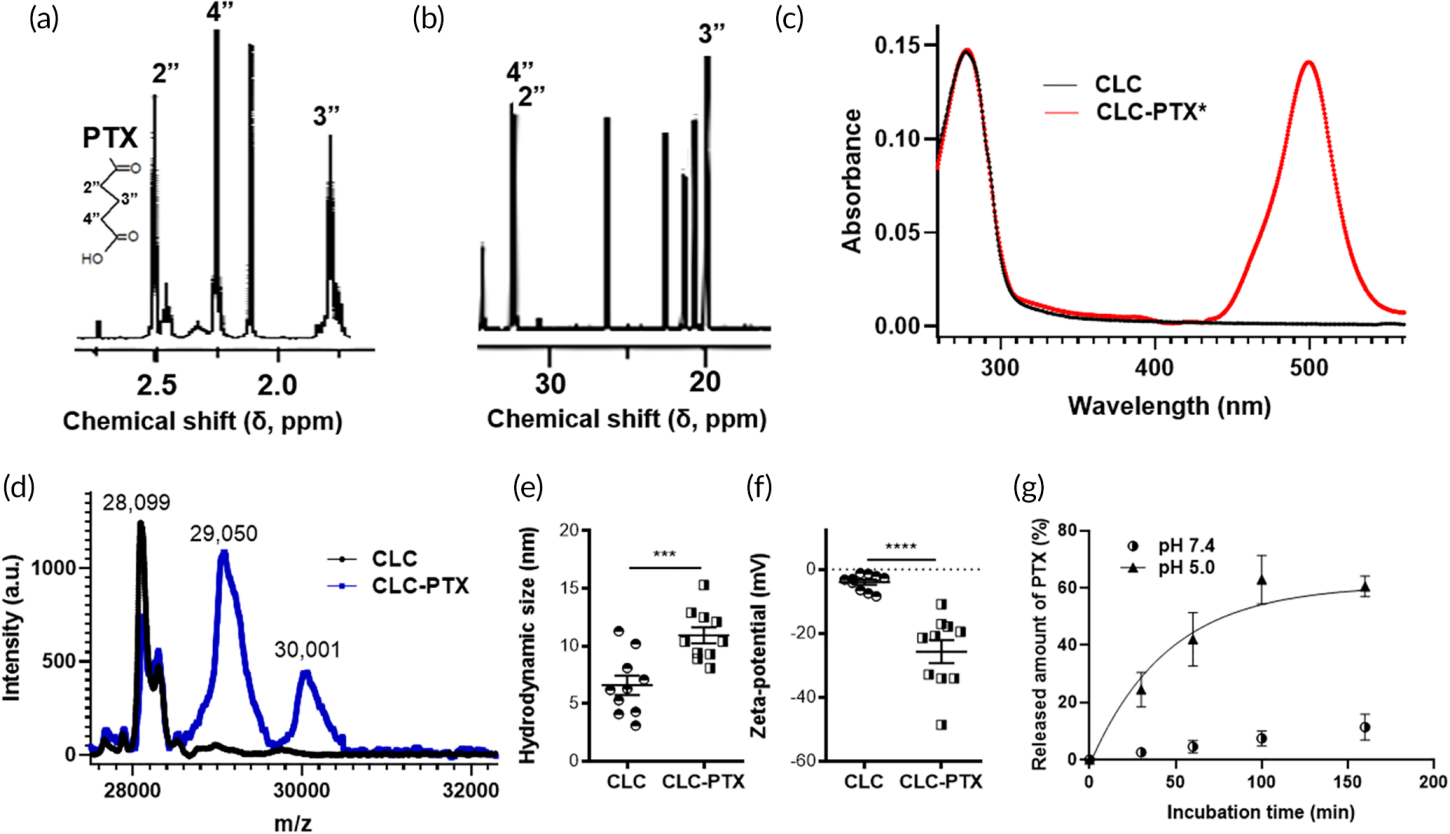
Characterization of clathrin light chain–paclitaxel (CLC‐PTX) conjugate. (a) ^1^H‐NMR (500 MHz, DMSO‐*d*_6_) spectrum of 2'‐glutaryl PTX. Chemical shifts; δ2.45 for 2'', δ1.75 for 3'', and δ2.23 for 4''. Inset: 2'‐glutaryl PTX. (b) ^13^C‐NMR (125 MHz, DMSO‐*d*_6_) spectrum of 2'‐glutaryl PTX in DMSO‐*d*_6_. Chemical shifts; δ32.3 for 2'', 19.9 for 3'', and δ32.5 for 4''. (c) UV–Vis absorption spectrum of CLC before and after conjugation with PTX*. (d) Matrix‐assisted laser desorption ionization time‐of‐flight (MALDI‐TOF) spectrum of CLC and CLC‐PTX conjugate. (e,f) Hydrodynamic size (e) and zeta‐potential (f) of CLC and CLC‐PTX in PBS. Student t‐test. (g) Release kinetics of PTX* from CLC in acetate buffer (pH 5.0) or phosphate buffer (pH 7.4)

CLC‐6 histone (MW ~28 kDa) was expressed in *Escherichia coli* (*E. coli*), confirmed by sodium dodecyl sulfate‐polyacrylamide gel electrophoresis (SDS‐PAGE), and extracted at a purity of >85%, as demonstrated by Western blot (data not shown). PTX is a chemotherapeutic drug used for the treatment of numerous cancers.^23^, ^24^, ^25^, ^26^, ^27^, ^28^ The use of PTX has several drawbacks, such as poor solubility and tissue toxicity^23^ that render it a prime candidate for drug‐carrier conjugation. Therefore, we used a pH‐sensitive linker to conjugate CLC to PTX (Figure S1). We conjugated CLC with PTX via esterification of C‐2′ in PTX by glutaric anhydride, a step that removes the cytotoxicity of PTX.^29^ We confirmed the synthesis of 2'‐glutaryl PTX by ^1^H‐NMR (Figure 1a) and ^13^C‐NMR (Figure 1b). In the acidic microenvironment of the tumor or the lysosomes of cancer cells, this ester bond in CLC‐PTX can be hydrolyzed, resulting in therapeutic activation of PTX.

Commercially available fluorescent PTX (Oregon Orange‐tagged PTX, abbreviated as PTX*) was used to confirm the conjugation of PTX to CLC. The maximal absorbance of PTX* is observed at a wavelength of around 500 nm. We conjugated 2′‐glutaryl PTX* to CLC via 1‐ethyl‐3‐(3‐dimethylaminopropyl)carbodiimide (EDC)/sulfo‐*N*‐hydroxysuccinimide (NHS) coupling to produce CLC‐PTX*, which was verified by ultraviolet–visible (UV–Vis) spectroscopy (Figure 1c). The absorbance of CLC‐PTX* was compared to unconjugated CLC, revealing absorption in the visible wavelength region, originating from PTX*. The ratio of PTX* to CLC was 1.1 ± 0.2, based on the extinction coefficients (ε) of CLC (λ = 280 nm) and PTX* (λ = 500 nm). The drug to antibody ratio (DAR) of PTX to CLC was confirmed again as 1.0 ± 0.1 by matrix‐assisted laser desorption ionization time‐of‐flight (MALDI‐TOF), a similar value as above (Figure 1d). After conjugation of PTX, the hydrodynamic size of CLC in phosphate‐buffered saline (PBS) increased from 6.6 ± 2.6 to 10.9 ± 2.2 nm (Figure 1e), whereas the zeta‐potential of CLC changed from −4.0 ± 2.6 mV to −25.7 ± 11.3 mV, presumably by the formation of an amide bond from a primary amine on CLC (Figure 1f). Next, CLC‐PTX* conjugates released PTX* at a higher rate over time in pH 5.0 acetate buffer in comparison to pH 7.4 phosphate buffer at 37°C (Figure 1g). Intravenous (iv) administration of CLC twice per week for 2 weeks to mice resulted in no notable toxicity to the lungs, liver, kidneys, or heart, as determined by histological observation (Figure S2A). In addition, no significant increase in serum creatinine and blood urea nitrogen (BUN), serologic markers of kidney injury, was noted in mice following treatment with CLC (Figure S2B and C).

### 2.2 Depletion of HSP70 in 4T1 cells hinders the uptake of CLCs

**FIGURE 2:**
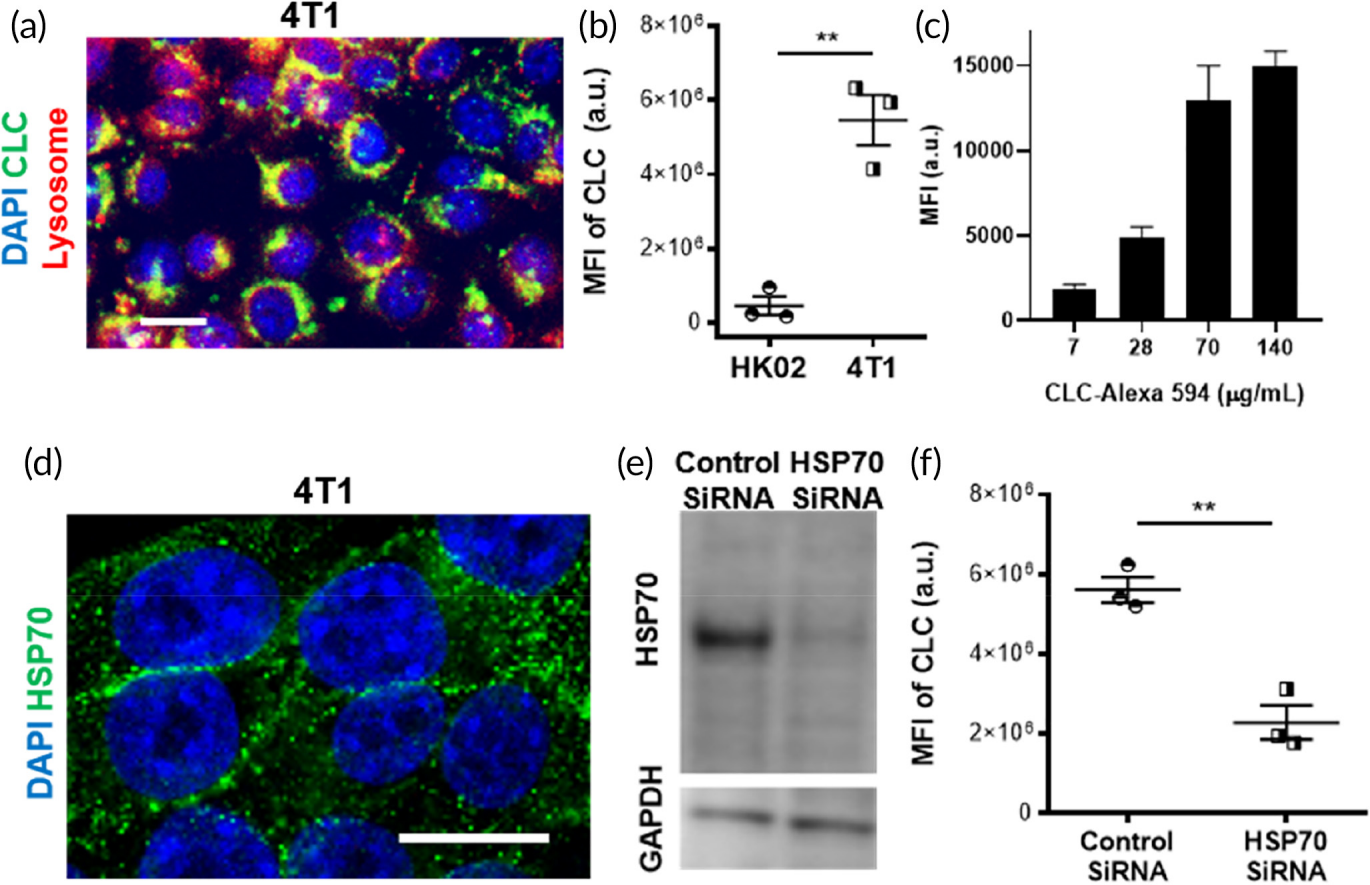
HSP70‐dependent accumulation of clathrin light chain (CLC) in 4T1 cancer. (a) Localization of CLC in the lysosome. 4T1 cells were stained with CLC antibody (green) and lysosome tracker (red), and counterstained with nuclear marker diamidino‐2‐phenylindole (DAPI) (blue). Scale bar: 25 μm. (b) CLC signal interacted with HK02 cells or 4T1 cells. (c) Correlation between concentration of CLC‐Alexa594 administered to 4T1 and its fluorescent signal on their surface. (d) Confocal fluorescence micrograph shows the expression of heat shock protein 70 (HSP70) on the surface of 4T1 cells. Scale bar: 25 μm. (e) Western blot of HSP70 expression by 4T1 cells following treatment with control siRNA or HSP70 siRNA. (f) CLC signal interacted with 4T1 cells with or without knockdown of HSP70 by siRNA. Significance was determined by Student's t‐test. The data are represented by means ± SD (**p < 0.01)

We sought to determine whether CLC was internalized by 4T1 mouse breast cancer cells by incubating these cells with CLCs attached to Alexa Fluor™ 488 dye. The colocalization of the CLCs with a lysosome marker was confirmed (Figure 2a), which indicated that the CLCs underwent endocytosis and were shuttled to acidic lysosomes in the 4T1 cells.

A virtual screening between CLC and a protein data bank (PDB) was conducted, resulting in the identification of three proteins with high binding energy (Table S1). Interestingly, one of these putative binding partners was HSP70, commonly reported as a cancer marker. A number of studies indicate the expression of HSP70 on the surface of cancer cells.^30^, ^31^, ^32^, ^33^, ^34^, ^35^ We compared the interaction with CLC in 4T1 cells and HK02 kidney tubular epithelial cells. 4T1 cells interacted with CLC 11.7‐fold more than HK02 cells (Figure 2b). We stained the surface of 4T1 cells with CLC‐Alexa 594. Fluorescence‐activated cell sorting (FACS) analysis revealed a positive correlation between the concentration of CLC‐Alexa 594 and the binding of CLC to the surface of the 4T1 cells (Figure 2C and S3). The mean fluorescence intensity (MFI) of CLC‐Alexa 594 was 51.8% of the MFI of an antibody against HSP70 (anti‐HSP70) in the 4T1 cells (Figure S4). 4T1 cells interacted with CLC‐Alexa 594 over time in vitro, resulting in a gradual increase of fluorescence, which eventually reached saturation (Figure S5A). The staining of 4T1 cells with anti‐HSP70 confirmed that these cells expressed HSP70 along their surface (Figure 2d). Similarly, the expression of HSP70 was detected on the surfaces of B16 cancer cells (Figure S5B). Expressions of HSP70 were higher in the cancer cell lines of 4T1 and B16 than the nonmalignant HK02 human kidney cell line (Figure S5C). We investigated the effect of HSP70 on the targeting capacity of CLC to 4T1 cells by silencing its expression with HSP70 siRNA in these cells. Successful knockdown of HSP70 in this condition was confirmed by Western blot (Figure 2e). The amount of CLC interacted with these HSP70 deficient 4T1 cells was 2.4‐fold lower (Figure 2f), suggesting that HSP70 plays a role in the uptake of CLC by 4T1 cells. We used bovine serum albumin (BSA) as a control to rule out the possibility of nonspecific uptake of CLC by 4T1 cells. We found that 4T1 cells internalized CLC‐Alexa 594 significantly more robustly in vitro than BSA‐Alexa 594 (Figure S5D).

### 2.3 CLCs localize to 4T1 breast primary tumor and metastatic lesions in mice

**FIGURE 3:**
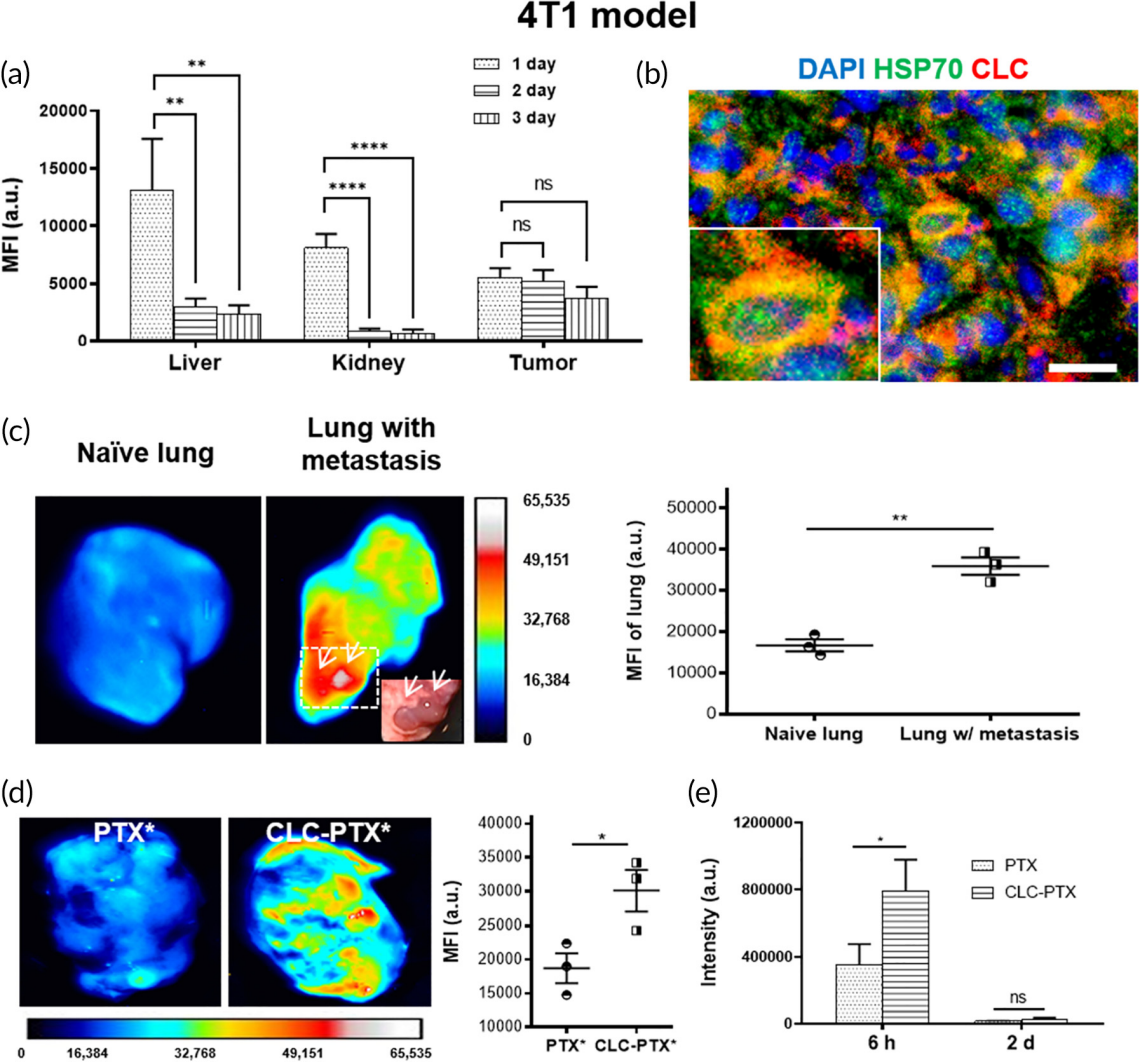
Biodistribution of clathrin light chain (CLC) in 4T1 tumor‐bearing mice. (a) A histogram of mean fluorescence intensity (MFI) in each organ over time. Tests were conducted by one‐way ANOVA with Holm–Sidak's post hoc. The data are represented by mean ± SD (*n* = 3, ^ns^ *p* > 0.05, **p < 0.01, and ****p < 0.0001). (b) Colocalization of heat shock protein 70 (HSP70) (green) and CLC (red) in 4T1 tumor. Inset: Magnified cell. Scale bar: 50 μm. (c) Ex vivo fluorescence images of lungs in naïve Balb/c mouse (left) and mouse 4T1‐bearing mouse (middle) after injection of CLC‐IR800. Arrow; metastatic foci. MFI of naïve or lung with metastasis (right). Student t‐test. (d) Enhanced targeting efficacy of PTX by CLC in 4T1 tumor. Left: Fluorescence micrograph 1 day after the treatment of free PTX* or CLC‐PTX* in BALB/c mice bearing 4T1 tumor. Right: Comparison of fluorescence intensity of PTX* and CLC‐PTX* in 4T1 tumor. Student t‐test. (e) Presence of PTX in serum after injection of free PTX or CLC‐PTX to BALB/c mice (Student t‐test, ^ns^ *p* > 0.05, *p < 0.05)

We examined the capacity of CLCs to localize to 4T1 breast cancer in vivo by administering CLC‐IR800 (4 mg/kg) *iv* to 4T1 tumor‐bearing BALB/c mice. The mice were euthanized at 1 day (1 d), 2 days, and 3 days following administration of CLCs, at which time points we measured the fluorescent signal of CLC‐IR800 in various organs, using an iBox Explorer^2^ Imaging Microscope. CLC‐IR800 signal in the tumor remained stable from 1 day to 3 days, but CLC signals in other organs faded out by 3 days (Figure 3a). The signals from lung and spleen were trivial for 3 days: CLC in these organs were also cleaned out (data not shown). We administrated CLC‐IR800 (4 mg/kg) to 4T1 tumor‐bearing BALB/c mice. After 6 h, we harvested the 4T1 tumors and stained them for HSP70. We confirmed a high overlap between the CLC and HSP70 signals (Figure 3b). We also conjugated CLC with PTX*, then injected 4T1 bearing mice *iv* with CLC‐PTX* and quantified the fluorescence of PTX*. PTX* did not clear significantly from the tumor within the span of the study, whereas the PTX* signal declined in the liver and the kidney over time (Figure S6).

The lung is the most common site of metastasis for breast cancer. Lung with 4T1 metastasis showed enhanced targeting signals of CLC as compared with naïve lung (Figure 3c). Interestingly, we observed that CLC‐IR800 localized more robustly to metastatic nodules of the lung (Figure 3c, inset), supporting the concept that CLC can deliver drugs specifically to metastatic lung lesions.

We further tested whether CLC‐PTX* localized to 4T1 breast cancer in vivo following *iv* administration to 4T1 breast tumor‐bearing BALB/c mice. Ex vivo fluorescent images of the tumors were acquired 1 day following administration of PTX* or CLC‐PTX* at an equivalent PTX* dose of 0.5 mg/kg, which showed that the accumulation of CLC‐PTX* in the tumor was higher than free PTX* (Figure 3D).

Finally, either PTX or CLC‐PTX was injected *iv* into BALB/c mice. At 6 h and 2 d, the sera of these mice were collected, and high‐performance liquid chromatography (HPLC) quantified PTX. We detected significant amounts of PTX in the sera of the CLC‐PTX group as compared with the free PTX group at 6 h, indicating that conjugation to CLC enhanced the blood circulation of PTX (Figure 3e). The PK of CLC‐PTX* derived from the fluorescence signal also revealed that conjugation with CLC prolonged the circulation of PTX (Figure S7).

### 2.4 CLC‐based delivery of PTX enhances its treatment efficacy for 4T1 breast cancer

**FIGURE 4:**
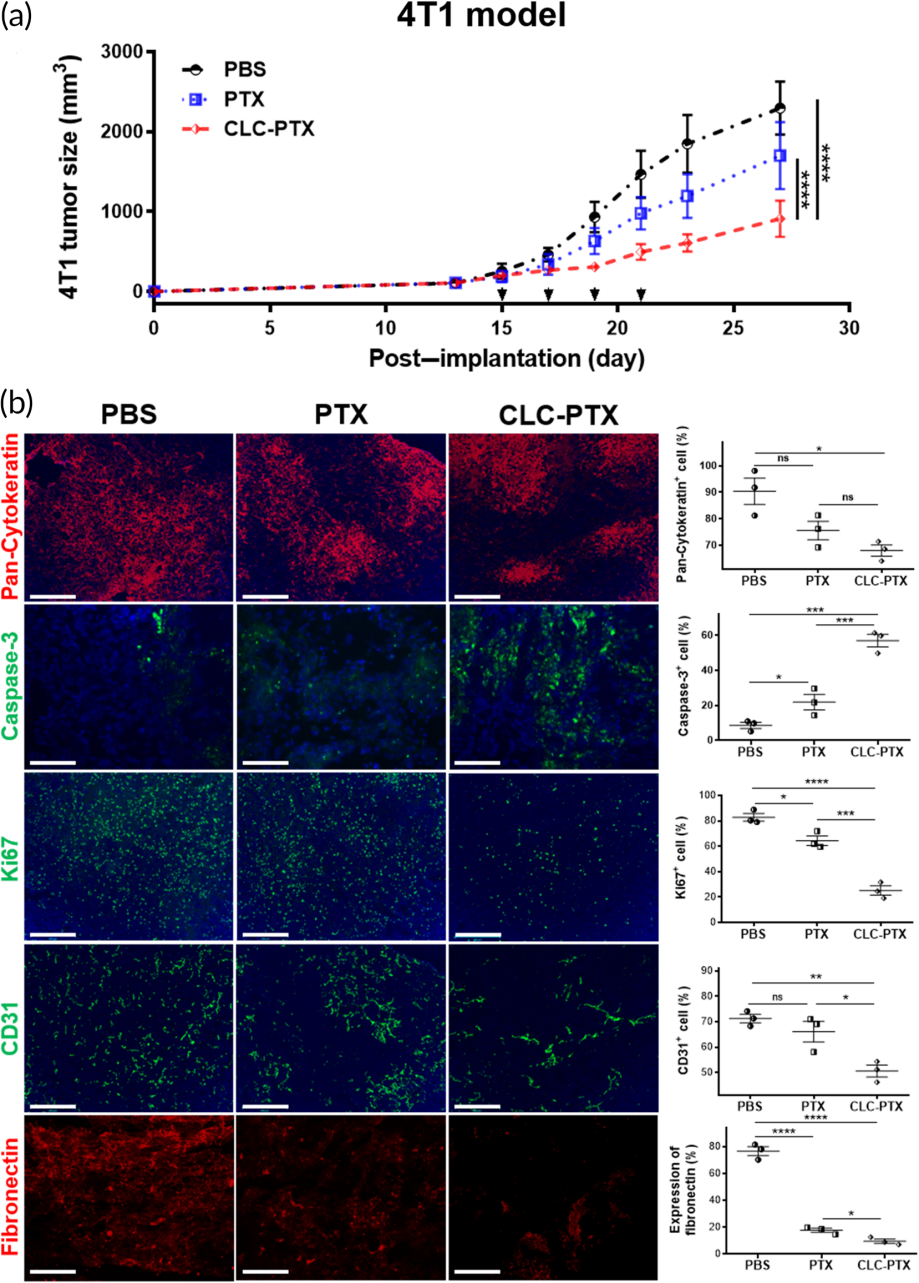
Inhibition of cancer growth in 4T1 murine breast cancer model by clathrin light chain–paclitaxel (CLC‐PTX). (a) Growth curves of 4T1 tumor after implantation in mice and treatment with free PTX, CLC‐PTX, or PBS. Treatment was started on d 13, and drugs were administered twice per week for 2 weeks (*n* = 6/group). At Day 27, all the mice were euthanized because the diameter of the tumors in the PBS‐treated mice reached ~2 cm. Arrows: treatment days. The data are represented by means ± SD (****p < 0.0001, the significance was determined by two‐way ANOVA with Holm–Sidak's post hoc. (b) Fluorescence micrographs of tumors at the end of treatment with PBS (first column), free PTX (second column), and CLC‐PTX (third column). Tumor sections were stained with antibodies to pan‐cytokeratin (first row), caspase 3 (second row), ki67 (third row), CD31 (fourth row), and fibronectin (fifth row). The fluorescence intensities from the fluorescence micrographs were compared in histograms (fourth column). Scale bar: 100 μm. The data were tested by one‐way ANOVA with Holm–Sidak's post hoc. The data are represented by means ± SD (*n* = 3, ^ns^ *p* > 0.05, *p < 0.05, **p < 0.01, ***p < 0.001, and ****p < 0.0001)

Therapeutic effects of CLC‐PTX conjugates were studied in BALB/c mice bearing 4T1 breast cancer, which is refractory to antineoplastic agents.^36^, ^37^ At 13 days following implantation of 1 × 10^5^ 4T1 cells—the timepoint when the tumor volume reached ~100 mm^3^—the mice were divided randomly into three groups. Each group received either PBS, free PTX, or CLC‐PTX twice per week *iv* for 2 weeks (equivalent amount of PTX, 8 μg/kg/injection). The tumors in the CLC‐PTX group displayed slower tumor progression in comparison to the two other treatment groups (Figure 4a). The tumor growth inhibition (*TGI*, as defined in Section 4) rate was over twofold higher in mice treated with CLC‐PTX, as compared with those treated with an identical dose of free PTX (60.7 ± 3.1 vs. 26.2 ± 2.1). The expression of the cancer marker pan‐cytokeratin was also inhibited by treatment with CLC‐PTX as compared to other groups (first row in Figure 4b). Expression of caspase‐3 by apoptotic cells in the 4T1 tumor was higher in the CLC‐PTX‐treated mice than as compared to other groups (second row in Figure 4b). In addition, 4T1 tumors treated with CLC‐PTX contained significantly fewer Ki67^+^ proliferating cancer cells in comparison to other treatment groups (third row in Figure 4b). Moreover, the 4T1 tumors treated with CLC‐PTX showed the lower CD31 and fibronectin in the 4T1 compared with the other two groups (fourth row in Figure 4b).

### 2.5 CLC‐PTX therapy disrupts metastasis of 4T1 breast cancer to the tumor‐draining lymph node and lung

**FIGURE 5:**
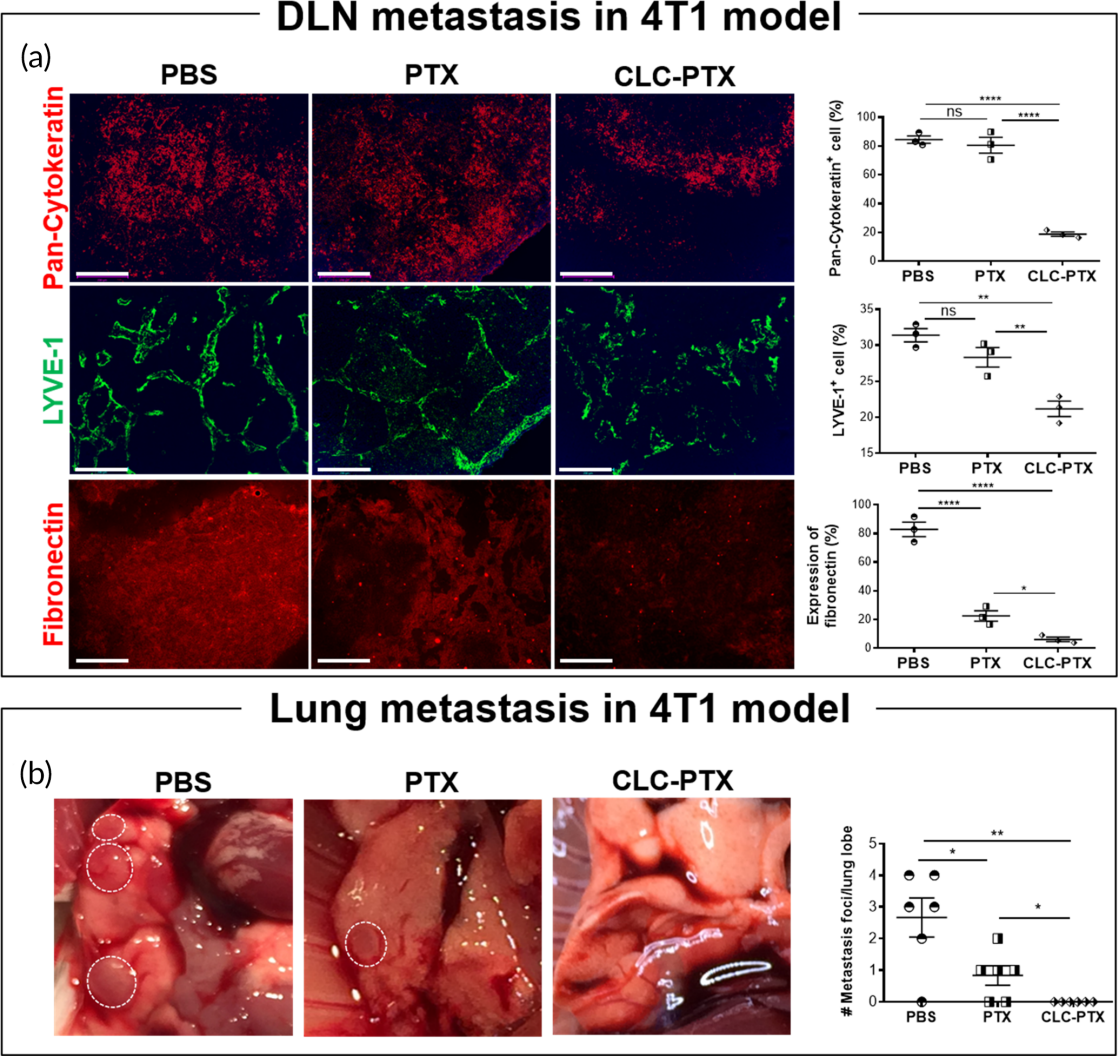
Inhibition of metastasis of 4T1 cancer to TDLN and lung by clathrin light chain–paclitaxel (CLC‐PTX). (a) Fluorescence micrographs for the tumor‐draining lymph node (TDLN) at the end of treatment (27 days postimplantation) with PBS (first column), free PTX (second column), and CLC‐PTX (third column). TDLN sections were stained with antibodies to pan‐cytokeratin (first row), LYVE1 (second row), and fibronectin (third row). Scale bar: 100 μm. (b) Representative photographs of the lung in each treatment group. Circle: Foci. Right graph; Number of metastatic foci per lung lobe. The data represent mean ± SD. All data were tested by one‐way ANOVA with Holm–Sidak's post hoc or Student t‐test (*p < 0.05, **p < 0.01, ***p < 0.001, and ****p < 0.0001)

We examined the TDLNs (inguinal lymph nodes) of 4T1 tumor‐bearing mice at 27 days following implantation (Figure 5). The expression of pan‐cytokeratin in the TDLN was significantly reduced by treatment with CLC‐PTX, as compared to the groups that received either PTX alone or no treatment (first row in Figure 5a). A similar decrease was also noted in the expression of LYVE‐1^+^ lymphatic vessels and fibronectin in the TDLNs of the CLC‐PTX‐treated group in comparison to other groups (second and third rows in Figure 5a). We also counted the number of visible metastatic nodules of lungs of treated groups that showed no pulmonary nodules in the CLC‐PTX group unlike other groups (Figure 5b).

### 2.6 CLC‐PTX therapy boosts growth inhibition of B16 melanoma

**FIGURE 6:**
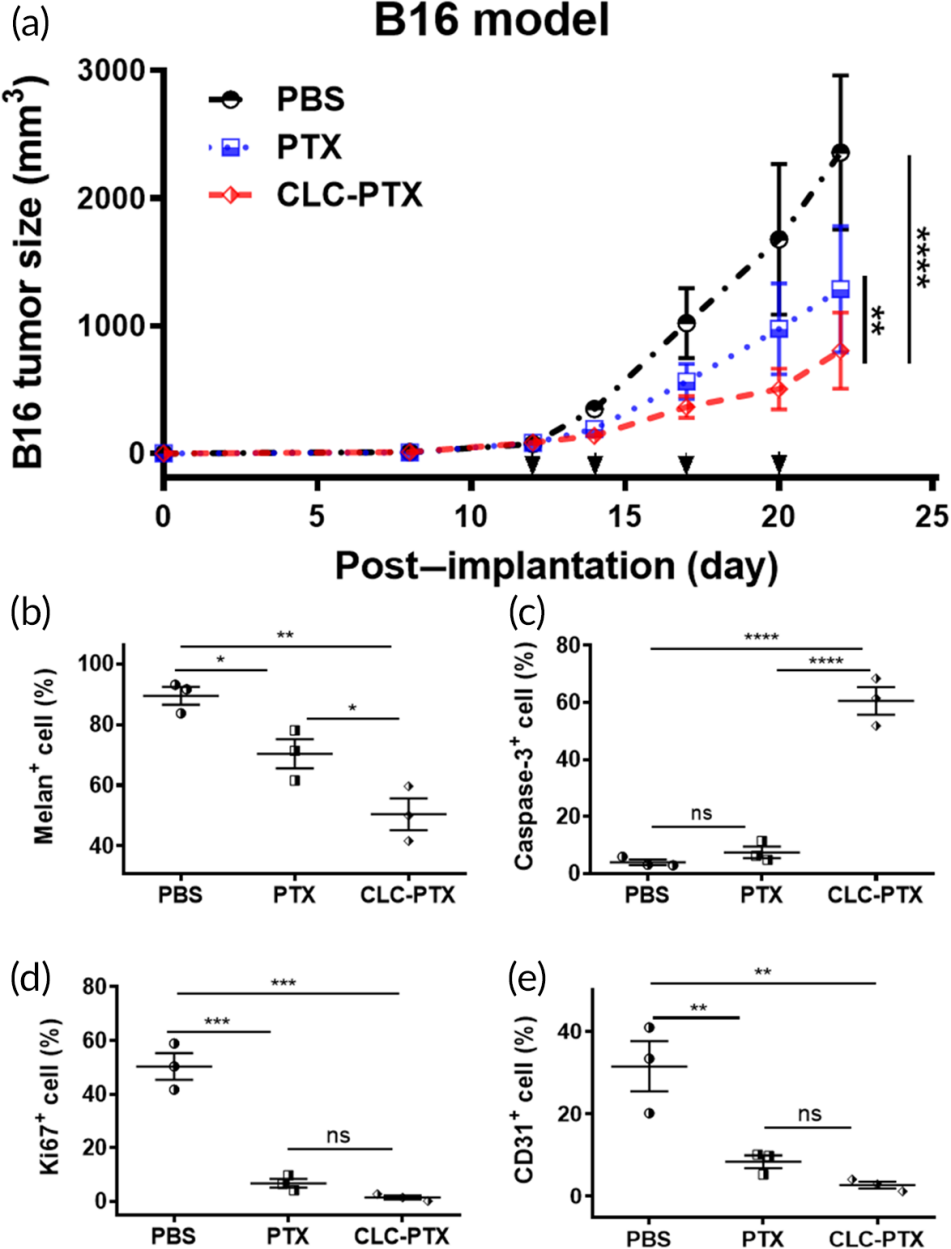
Inhibition of cancer growth in B16 murine melanoma model by clathrin light chain–paclitaxel (CLC‐PTX). (a) Growth curves of B16 tumor after implantation in mice and treatment with free PTX, CLC‐PTX, or PBS. Treatment was started on 12 days postimplantation, and drugs were administered twice per week for 2 weeks (*n* = 6/group). Arrows: treatment days. The data are represented by mean ± SD (**p < 0.01 and ****p < 0.0001, the significance was determined by two‐way ANOVA with Holm–Sidak's post hoc. (b–e) Percentage of positive cells of melan‐A (b), caspase‐3 (c), Ki67 (d), and CD31 (e) analyzed in immunofluorescence staining of B16 tumor tissues. The data were tested by one‐way ANOVA with Holm–Sidak's post hoc. The data are represented by means ± SD (n = 3, ^ns^ *p* > 0.05, *p < 0.05, **p < 0.01, ***p < 0.001, and ****p < 0.0001)

We also investigated the antineoplastic efficacy of CLC‐PTX in a B16 melanoma mouse model, using C57BL/6 mice as the host. Treatments were administered after the tumor size reached ~100 mm^3^, following a schedule and dosing identical to the 4T1 study above. Again, CLC‐PTX displayed the most potent inhibitory effect on tumor growth: at the end of the study, the tumors of the mice treated *iv* with CLC‐PTX were the smallest (0.81 ± 0.30 × 10^3^ mm^3^), as compared to the PBS‐treated mice (2.36 ± 0.61 × 10^3^ mm^3^) and free PTX‐treated mice (1.29 ± 0.50 × 10^3^ mm^3^) (Figure 6a). *TGI* by CLC‐PTX was higher than free PTX (68.6 ± 4.0% vs. 47.3 ± 2.5%, **p < 0.01). Immunostaining demonstrated a decline of Melan‐A^+^ cells with an increase in caspase‐3 expression in the CLC‐PTX‐treated mice as compared to other groups (Figure 6b,c). The B16 tumors in mice treated with CLC‐PTX also contained fewer CD31^+^ blood vessels and Ki67^+^ cells (Figure 6d,e).

### 2.7 CLC‐based delivery augments the therapeutic efficacy of PTX for lung carcinoma

**FIGURE 7:**
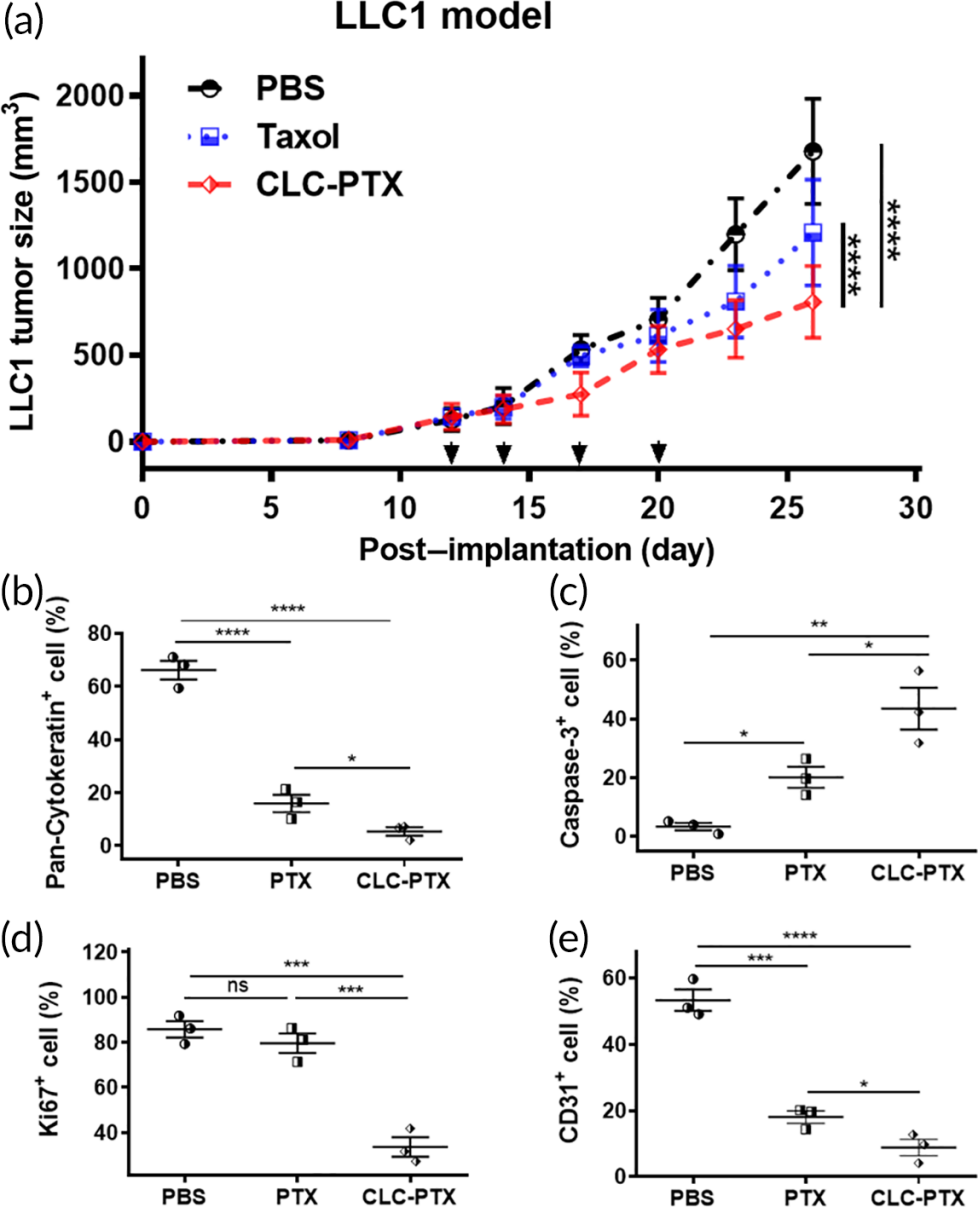
Inhibition of cancer growth in LLC1 murine lung carcinoma model by clathrin light chain–paclitaxel (CLC‐PTX). (a) Growth curves of LLC1 tumor after implantation in mice and treatment with free PTX, CLC‐PTX, or PBS. Treatment was started on 12 days postimplantation of tumor, and drugs were administered twice per week for 2 weeks (*n* = 6/group). Arrows: treatment days. The data are represented by mean ± SD (****p < 0.0001, the significance was determined by two‐way ANOVA with Holm–Sidak's post hoc. (b–d) Rate of cells stained with antibodies to pan‐cytokeratin (b), caspase‐3 (c), Ki67 (d), and CD31 (d). The data were tested by one‐way ANOVA with Holm–Sidak's post hoc. The data are represented by means ± SD (*n* = 3, ^ns^ *p* > 0.05, *p < 0.05, **p < 0.01, ***p < 0.001, and ****p < 0.0001)

Therefore, we administered CLC‐PTX *iv* to C57BL/6 mice bearing LLC1 murine lung carcinoma, following a schedule identical and dosing in the aforementioned tumor models. The tumors in the CLC‐PTX group (0.80 ± 0.21 × 10^3^ mm^3^) were significantly smaller than those in the mice that received PBS (1.68 ± 0.30 × 10^3^ mm^3^) and free PTX (1.21 ± 0.31 × 10^3^ mm^3^) (Figure 7a). In addition, *TGI* by CLC‐PTX was higher than free PTX (56.2 ± 4.3% vs. 30.3 ± 2.7%, **p < 0.01). Next, we stained LLC1 tumor tissue sections with the cancer marker pan‐cytokeratin, apoptosis marker caspase‐3, proliferation marker Ki67, and vascular marker CD31, and examined them by fluorescence microscopy. Treatment with CLC‐PTX reduced the amount of Pan‐Cytokeratin^+^ cancer cells significantly, whereas Caspase‐3^+^ apoptotic cells increased (Figure 7b,c). Consistent with these results, the proliferation of LLC1 cancer cells was also blocked critically by treatment with CLC‐PTX in comparison to free PTX (Figure 7d). Furthermore, the LLC1 tumors in mice treated with CLC‐PTX contained the lowest density of vascular structures (Figure 7e). Thus, targeted treatment of LLC1 lung cancer with CLC‐PTX inhibited growth, enhanced apoptosis, hindered proliferation, and reduced vascularization of LLC1 lung carcinoma.

## 3 DISCUSSION

Targeted drug delivery systems have attracted major interest and already entered into clinical practice for various diseases, but their application to cancer remains to be developed.^38^, ^39^, ^40^ Although chemical linkers in ADCs can be noncleavable, the majority of ADCs in clinical development have specific release mechanisms that permit the controlled release of the drugs at target sites.^41^ For example, acid‐cleavable linkers harness the acidity within lysosomes (pH 4.5–5.0). This strategy has already yielded clinical success, as demonstrated by the products Mylotarg®^42^ and Besponsa®.^43^ Esterification by anhydrides is a well‐established method for drug release in the field of medicine.^44^, ^45^ Here, we used glutaric anhydride to add hydrolytically cleavable functions to CLC‐based conjugates. Our conjugation method capitalizes on the obligate localization of CLC‐PTX to the lysosomes as the basis for the release of PTX from CLC, activating its therapeutic function. Moreover, the small size of ADCs is critical for optimal tumor penetration. Currently, antibody fragments have been engineered, such as nanobodies, diabodies, and single‐domain antibodies, to overcome the limitations of using large IgG antibodies (~150 kDa).^6^, ^46^, ^47^ The small size of CLC can confer advantages with respect to tumor penetration in the absence of fractionation. Since CLC is a protein that is widely distributed in the human body,^48^ it may have a lower toxicity profile. In addition, large‐scale production of CLC using an expression system increases its utiltiy.^49^

Metastasis is the primary factor of cancer morbidity and mortality.^50^, ^51^, ^52^ Metastatic breast cancer presents a major clinical challenge, causing many deaths on a yearly basis.^53^ Beyond current antibody‐based treatments,^54^, ^55^ the development of other nonantibody modalities for targeted therapy is required to improve breast cancer treatment. A previous preclinical approach to target HSP70 expressed by cancer cells for in vivo tumor imaging has yielded success.^56^ Vesicles coated with clathrin triskelia are critical for membrane trafficking in cells. A clathrin triskelion is composed of three heavy chains and three light chains.^11^ Notably, the light chain acts as a regulatory unit in this triskelion through its binding with calcium ion^13^ or calmodulin,^14^ as well as its phosphorylation.^15^ Importantly, HSP70 participates in one such regulatory mechanism, as it interacts directly with CLC to disassemble clathrin‐coated vesicles. HSP70 targets a specific region of CLC, which is known as the “HSP70 interaction sequence,” identified by anti‐peptide antibodies.^57^ Other studies have reported high expression of HSP70 in breast cancer,^58^ melanoma,^33^ and lung carcinoma.^56^ HSP70 is expressed generally on the plasma membranes of primary tumor cells and distant metastases.^30^ This membrane‐bound HSP70 has been identified in a variety of different primary cancers.^31^, ^32^ Moreover, the density of membrane‐bound HSP70 in metastatic lesions is higher than the corresponding primary tumors in mouse and human cancer models.^33^, ^34^, ^35^ Expression of HSP70 increases further in high‐grade cancers, correlating with enhanced motility, invasion, and metastasis.^59^

Nonetheless, future in vivo targeting studies are required to define the mechanisms by which HSP70 functions as a target of CLC‐conjugated drug delivery. In addition, HSP70 produced by tumor cells may undergo differential glycosylation, so kinetic studies tailored to each type of cancer may be required for more specific and accurate assessment of the binding affinity of HSP70 to CLC. The performance of a mutational study to identify putative binding site of CLC^57^ is also required in the future to characterize thoroughly the interaction between CLC and HSP70.

Our data suggest that CLC targeted the primary tumor and metastatic lung lesions of 4T1 breast cancer in mice with high efficacy. While there was an increase in CLCs in the peripheral organs, in particular, the kidney; however, this retention faded after 3 days. Nonetheless, the tumor uptake of exogenously administered CLCs remains relatively stable over time.

CLC‐PTX enhanced the therapeutic efficacy of PTX on the primary 4T1 tumor, as demonstrated by suppression of tumor growth, enhancement of cancer cell apoptosis, and especially inhibition of angiogenesis. CLC‐PTX treatment was also found to reduce the density of lymphatic vasculature, which often contributes to the immunosuppressive environment of TDLN. Concomitantly, distant lung metastasis was also inhibited more effectively by treatment with CLC‐PTX than free PTX. Excessive accumulation of extracellular matrix (ECM) in the tumor stroma is referred to as the desmoplastic reaction, commonly reported as a major obstacle to the treatment of cancers.^60^, ^61^, ^62^ These ECM fibers facilitate angiogenesis through their production of growth factors and chemokines.^61^ Furthermore, increased density of ECM lessens tissue rigidity and escalates interstitial fluid pressure, resulting in reduced drug penetration.^63^, ^64^ The TDLN is the main secondary lymphoid organ in which the immune response to the tumor is generated and regulated. TDLNs are the first location for metastasis of the primary tumor; therefore, the spread of cancer to the TDLN correlates with a poor prognosis. Fibronectin staining indicated that CLC‐PTX treatment blocked the formation of excessive ECM in both the primary 4T1 tumor and TDLN significantly, suggesting that CLC‐based drug conjugates render the ECM environment less favorable for cancer growth through their concentration of the chemotherapeutic drug at the cancer site. We noted similar therapeutic effects on other tumor types as well. Targeted ADCs have been developed to treat melanoma, including CDX011‐05^65^ and DEDN6526A,^66^ both of which are currently under clinical trials. CLC‐PTX boosted the suppression of B16 tumor by PTX, as confirmed by the triad of higher cancer cell apoptosis, lower cancer cell density, and inhibition of angiogenesis. CLC‐PTX significantly alleviated lung carcinoma growth in comparison to free PTX, as demonstrated by diminished cancer cell density, higher apoptosis, and lower fibrosis. Since a recent study demonstrated substantial heterogeneity in the expression of cancer markers such as HER2^67^, ^68^ in metastatic lesions, our CLC‐based therapeutics may result in more uniform targeting of all HSP70‐expressing metastatic lesions.

## 4 MATERIALS AND METHODS

### 4.1 Cell lines and tissue

4T1 mouse breast cancer (ATCC® CRL‐2539™), B16 mouse melanoma (ATCC® CRL‐6322™), LLC1 mouse lung carcinoma (ATCC® CRL‐1642™), and HK02 human kidney tubular epithelial cell lines (ATCC® CRL‐2190™) were purchased from American Type Culture Collection (ATCC, VA, USA). 4T1, B16, LLC1, and HK02 cells were cultured in RPMI‐1640 or Dulbeccos modified Eagles medium with 10% fetal bovine serum and 1% penicillin/streptomycin (pen/strep).

### 4.2 Mice

C57BL/6 (JAX# 000664) and BALB/c (JAX# 000651) mice were obtained from The Jackson Laboratories. All animal experiments and methods were performed in accordance with the relevant guidelines and regulations approved by the Institutional Animal Care and Use Committee (protocols: 2016 N000167/04977) of Brigham and Women's Hospital (Boston, MA).

### 4.3 Preparation of CLC

Human CLC was expressed and optimized for an *E. coli* expression system as follows. The human CLC gene was codon‐optimized for expression in *E. coli* and chemically synthesized by Biomatik. The synthesized gene was subsequently cloned into NdeI/HindIII sites with pET30a vector. These expression plasmids were used to transform the *E. coli* BL21(DE3). His‐tag was applied to a Ni‐NTA column and eluted in a buffer containing 10 mM Tris, 0.15 M NaCl, 8 M urea, and 0.3 M imidazole at a pH of 8.0.

Gene sequence; ATGGCGGAACTGGACCCGTTCGGCGCTCCGGCAGGCGCACCGGGCGGTCCGGCGCTGGGTAACGGCGTTGCGGGTGCTGGTGAAGAAGACCCGGCAGCAGCGTTCCTGGCGCAGCAGGAATCTGAAATCGCAGGTATCGAAAACGATGAAGCGTTCGCGATCCTGGACGGTGGTGCTCCGGGTCCGCAGCCGCACGGTGAACCGCCGGGTGGTCCGGATGCGGTTGACGGTGTTATGAACGGCGAGTACTACCAGGAGTCTAACGGTCCGACCGATTCTTACGCGGCAATTAGCCAGGTTGATCGTCTGCAATCCGAACCGGAATCTATCCGTAAATGGCGTGAGGAGCAGATGGAACGCCTGGAAGCTCTGGACGCGAACTCTCGCAAACAGGAGGCGGAATGGAAAGAAAAAGCGATCAAAGAGCTGGAAGAATGGTATGCGCGTCAGGACGAACAGCTGCAAAAAACCAAAGCGAACAACCGTGTGGCGGACGAAGCATTCTACAAACAGCCGTTTGCGGACGTTATCGGTTACGTTACCAACATCAACCATCCGTGCTACTCTCTGGAGCAGGCAGCGGAAGAAGCGTTCGTGAACGACATCGACGAATCTAGCCCAGGCACCGAATGGGAACGTGTTGCGCGCCTGTGCGACTTCAACCCGAAATCTTCTAAACAGGCTAAAGACGTTTCTCGTATGCGTTCTGTTCTGATCTCTCTGAAGCAGGCTCCGCTGGTTCAC.

Amino sequence; MAELDPFGAPAGAPGGPALGNGVAGAGEEDPAAAFLAQQESEIAGIENDEAFAILDGGAPGPQPHGEPPGGPDAVDGVMNGEYYQESNGPTDSYAAISQVDRLQSEPESIRKWREEQMERLEALDANSRKQEAEWKEKAIKELEEWYARQDEQLQKTKANNRVADEAFYKQPFADVIYVTNINHPCYSLEQAAEEAFVNDIDESSPGTEWERVARLCDFNPKSSKQAKDVSRMRSVLISLKQAPLVH.

The purity of CLC was >85%, as estimated by a Coomassie blue‐stained SDS‐PAGE gel. The concentration of CLC was determined by Bradford protein assay, using BSA as a standard. Its molecular weight was 28.1 kDa, and its isoelectric point was 4.37.

### 4.4 Virtual screening of binding candidates to CLC

First, the 3D structure of CLC was established, according to its amino acid sequence. Then, screening was performed in silico to find interaction partners in the PDB database. GOR IV was used for secondary structure analysis of the protein. The 3D structure of the protein was generated by Homology Modeling Program, developed by Profacgen. In total, 1590 human protein PDB entries were downloaded from www.rcsb.org. Three proteins with the highest binding energy were identified through AutoDock Vina: ADP‐ribosylation factor 3, HSP70, and serine/threonine‐protein phosphatase PP1‐beta catalytic subunit (Table S1). Among these, HSP70 has commonly been reported as a cancer marker.

### 4.5 Lysosome staining and uptake assay in 4T1 cells

CLC or BSA (10735078001; Sigma‐Aldrich) was labeled with Alexa Fluor™ 488 NHS Ester (Thermo Fisher Scientific) or Alexa Fluor™ 594 NHS Ester (Thermo Fisher Scientific) and incubated with 4T1 cells for 2 h at 37°C. After five washes with PBS buffer, 4T1 cells were stained with lysosomal staining kit (ab112137; Abcam). 4T1 cells were fixed with 4% paraformaldehyde (Electron Microscopy Sciences), and diamidino‐2‐phenylindole (DAPI) (VECTASHIELD, Vector Laboratories) was used to counterstain the cell nuclei. The cells were visualized using an EVOS™ FL Auto 2 Imaging System (Thermo Fisher Scientific). After splitting each color channel, the area with fluorescence was measured for each color channel with Image J (National Institutes of Health; Bethesda, MD). The percent of uptake of CLC was calculated by dividing with measured area from DAPI channel.

### 4.6 Synthesis of 2'‐glutrayl PTX and conjugation to CLC

Glutaric anhydride (100 mg, Sigma‐Aldrich) and PTX (33 mg, LC laboratories) were prepared in a 4 ml vial dried under high vacuum for 24 h and dissolved in 1 ml of pyridine. The solution was stirred at room temperature under Ar atmosphere for 2 h. The mixture was diluted with 300 μl of methanol, and 5 μl of solution was injected into liquid chromatography/mass spectrometery (LC/MS) (Agilent 1200, USA) with a gradient reversed‐phase system (10%–100% ACN/H_2_O with 0.1% formic acid for 20 min), using a Phenomenex Luna 5 μm C_18_ column (100 × 4.6 mm, flow rate 0.7 ml/min, UV 250 nm detection). The product was detected at 14.1 min, and molecular weight was confirmed as 969 g/mol by electron spay ionization mass spectrometry analysis ([M‐H]^−^ m/z at 968]). 2'‐Glutaryl PTX was purified by reversed‐phase HPLC (Phenomenex Luna 5 μm C_18_ 250 × 10.0 mm, flow rate 2 ml/min, UV 600 nm detection) with a gradient solvent system (15%–75% ACN/H_2_O with 0.1% formic acid for 40 min). The product was eluted at a retention time of 13.4 min under these HPLC conditions. 2′‐Glutaryl PTX was confirmed by ^1^H‐NMR (proton nuclear magnetic resonance) and ^13^C‐NMR (carbon nuclear magnetic resonance) spectra. 2'‐glutaryl PTX (0.2 mg) dissolved in dimethyl sulfoxide (DMSO) (Thermo Scientific Fisher) was activated with 1‐ethyl‐3‐(3‐dimethylaminopropyl)carbodiimide hydrochloride (EDC, 0.4 mg, Sigma‐Aldrich) and Sulfo‐NHS (N‐hydroxysulfosuccinimide) (1.1 mg, Thermo Scientific Fisher) for 15 min at RT in 2‐(N‐morpholino)ethanesulfonic acid (MES) buffer (pH 6.0, Thermo Scientific Fisher) (final solution; ~1 ml in 10% DMSO). The EDC was quenched by 2‐mercaptoethanol (1.4 μl, Sigma‐Aldrich) for 10 min. Immediately, the pH of the solution was increased by NaHCO_3_ (0.1 M, Sigma‐Aldrich) to ~8. CLC dissolved in PBS (pH 7.4, Corning) was mixed with the activated 2′‐glutaryl PTX for 2 h at RT (1:10 molar ratio of CLC to PTX, final solution; 10% DMSO). Dialysis was performed twice by a centrifugal filter (Amicon®, 10 kD MWCO; Sigma‐Aldrich) at 10,000 rpm for 15 min to remove the free PTX. The solution was purified further by a desalting column (Zeba™, 7 kD MWCO, Thermo Scientific Fisher).

### 4.7 Ratio of PTX* to CLC

Absorbance was measured by UV–Vis spectroscopy (VWR™, UV‐1600PC). The molar extinction coefficient (ε_280 nm_) of CLC was extracted from the absorption spectrum of CLC solutions at different concentrations. The ε_CLC,280 nm_ was 4.8 × 10^4^, whereas ε_PTX,500 nm_ was 4.2 × 10^4^. The concentration of CLC (C_CLC_) in the CLC‐PTX* solution was calculated by (A_CLC‐PTX*,280 nm_ – A_PTX*,280 nm_)/ε_CLC,280 nm_, and C_PTX*_ was by (A_CLC‐PTX*,500 nm_/ε_PTX*,500 nm_).

### 4.8 MALDI‐TOF measurement of CLC‐PTX

MALDI mass analyses of CLC and CLC‐PTX were performed with a Bruker MicroFlex™ MALDI‐TOF in negative mode. The protein solution was mixed with sinapinic acid matrix solution (6 mg/ml, 50:50 ACN:H_2_O, containing 0.1% v/v TFA) at 1:1 ratio. The 2 μl of the samples was spotted, air‐dried, and detected with a linear detector. The data were analyzed using Bruker Daltonics flexAnalysis. External calibration was performed with BSA with [M‐H] average mass (m/z) of 66,428.

### 4.9 Quantification of released amount of PTX* in acidic buffer

CLC‐PTX* (0.5 mg of CLC) was incubated in acetate buffer (10 mM, pH 5.0) or phosphate buffer (10 mM, pH, 7.4) at 37°C. The solutions were dialyzed with 5% DMSO solution, and filtrates were analyzed by UV–Vis spectroscopy to quantify the amount of free PTX* in comparison to the absorbance of the solution prior to incubation.

### 4.10 FACS analysis

The surface of 4T1 cells (1.0 × 10^5^) was stained with CLC‐Alexa 594 or anti‐HSP70‐Alexa 594 in FACS buffer (DPBS +2% fetal bovine serum +1 mM EDTA +0.1% sodium azide) at 4°C for 30 min. Cells were washed by FACS buffer and fixed with FACS buffer containing 1% formalin. The autofluorescence from the cell itself was unmixed and only single cells were gated (Cytek® Aurora). Analysis of flow cytometry data was performed by FlowJo software (FlwoJo LLC, Ashland, OR). The histogram over Alexa 594 channel was extracted and the total fluorescence signal was divided by the total number of the cell.

### 4.11 SiRNA transfection and Western blot

Cells were transfected with Lipofectamine 2000 (Invitrogen), according to the manufacturer's instructions. Briefly, cells were plated at 20%–30% density in 12‐well plates 24 h prior to transfection. For siRNA transfection, the equivalent of 200 nM of siRNA per well of a 12‐well plate was utilized. After a 48 h incubation period, the lysates of the cells were measured using the Bradford assay. Equal amounts of protein were separated by SDS‐PAGE and transferred to a polyvinylidene fluoride (PVDF) membrane. The membranes were immunoblotted with the following specific antibodies: anti‐HSP70, anti‐rabbit IgG‐HRP (Sigma‐Aldrich), anti‐rat IgG‐HRP (Sigma‐Aldrich), and anti‐GAPDH (Sigma‐Aldrich), using standard protocols. The blots were developed with West Dura chemiluminescent substrates using a Bio‐Rad ChemiDoc imaging system.

### 4.12 Ex vivo biodistribution of CLC

Animal studies were approved and conducted according to the Institutional Animal Care and Use Committee of Brigham and Women's Hospital, Boston, MA. CLC‐IR800 was prepared by incubating CLC with IRdye 800CW NHS ester (LI‐COR, 929‐70,020) for 2 h at RT. The crude was purified by a desalting column (Zeba™, 7 kD MWCO, Thermo Scientific Fisher). For ex vivo biodistribution studies, BALB/c mice were injected *s.c*. with 1.0 × 10^5^ 4T1 cells. At 14 days postimplantation, these mice received a single *iv* injection of CLC‐IR800 conjugate (4 mg/kg). At 1 day, 2 days, and 3 days following injection, the mice were euthanized via carbon dioxide inhalation and cervical dislocation, and their major organs were harvested and imaged by using a UVP iBOX Explorer Imaging Microscope (UVP) equipped with a 750–780 nm band‐pass excitation filter and an 800 nm long‐pass emission filter. MFI for each organ was measured using the region of interest (ROI) function of ImageJ (National Institutes of Health; Bethesda, MD).

### 4.13 Ex vivo tumor imaging of CLC‐PTX

CLC‐PTX* was prepared with the identical protocol for the synthesis of CLC‐PTX except the use of PTX* (Oregon Green™ Taxol, P22310, Thermo Fisher Scientific). BALB/c mice were *s.c*. implanted with 1.0 × 10^5^ 4T1 cells. At 14 days postimplantation, these mice received a single *iv* injection of CLC‐PTX* conjugate (at an equivalent PTX* dose of 0.5 mg/kg). After 1 day, the mice were euthanized via carbon dioxide inhalation and cervical dislocation, and their major organs were harvested and imaged by using a UVP iBOX Explorer Imaging Microscope (UVP) equipped with a 455–495 nm band‐pass excitation filter and a 513–557 nm band‐pass emission filter. MFI of the organs was measured following subtraction of autofluorescence, using the ROI function of ImageJ.

### 4.14 Pharmacokinetics of CLC‐PTX

BALB/c mice (female; 7–8 weeks) were injected intravenously with PTX or CLC‐PTX at an equivalent PTX dose of 2.5 mg/kg. Sera from three mice were collected at 6 h and 2 days following drug administration. The serum samples were stored at −20°C until analysis. Serum samples (200 μl) were mixed with acetate buffer (3 M, pH 5.0, 1 μl) for 1 day at 37°C. PTX in the serum was extracted by incubation with acetonitrile (6 ml) for 2 h at 37°C. After centrifugation (10 min, 5000 rpm), the supernatants were analyzed by HPLC. High‐resolution electrospray ionization mass spectra were acquired using Agilent LC‐q‐TOF Mass Spectrometer 6530‐equipped with a 1290 uHPLC system. The collected samples were injected into a reversed‐phase HPLC column (Kintex® C_18_: 100 × 2.1 mm, 2.6 μm) under the following gradient elution system: 10% acetonitrile/water to 100% acetonitrile/water for 20 min with a flow rate of 0.3 ml/min. Extracted ion is 854 m/z.

### 4.15 Pharmacokinetics of CLC‐PTX*

BALB/c mice (female; 7–8 weeks) were *iv* injected with PTX* or CLC‐PTX* at an equivalent PTX* dose of 0.5 mg/kg. Sera from three mice were collected at 6 h and 2 days following drug administration. The serum samples were stored at −20°C until analysis. The PTX plasma concentration was analyzed with the aid of the iBOX microscope.

### 4.16 Therapeutic studies in tumor‐bearing mice

BALB/c mice (female; 7–8 weeks) were implanted subcutaneously with 1.0 × 10^5^ 4T1 cells in the left fourth mammary gland. C57BL/6 mice (female; 7–8 weeks) were inoculated subcutaneously with 1.0 × 10^5^ B16, LLC1, or Pan02 cells in the right rear flanks. When the tumor size reached ~100 mm^3^, the mice were randomly divided into three groups (*n* = 6). All groups received treatments *iv*; the first group was injected with PBS (control), the second group with free PTX (total dose of PTX = 32 μg/kg), and the final group with CLC‐PTX (total dose of CLC‐PTX = 1.06 mg/kg, PTX dose identical to free PTX group). The treatment schedule consisted of twice‐per‐week injections for 2 weeks. The tumor size and body weight of the mice were monitored during the treatment course. The length (*l*) and width (*w*) of the tumor was measured by a digital Vernier caliper, and tumor volume (*V*) was defined as $V=l\times w^{2}/2$. We evaluated the *TGI* rate of each group ($\mathrm{TGI}\;\left(\%\right)=\left(V_{c}-V_{t}\right)/\left(V_{c}-V_{i}\right)\times 100$), in which *V*_*c*_ is the volume of the control tumor at the end of the study, *V*_*t*_ is the volume of drug‐treated tumor at the end of the study, and *V*_*i*_ is the volume of tumor at the initial treatment.

Before the diameter of the tumor reached ~2 cm, the mice were euthanized, and lungs, livers, kidneys, spleen, tumor, and draining inguinal lymph nodes were harvested and embedded in optimum cutting temperature (OCT) compound (Tissue Tek; Sakura Finetek; Torrance, CA).

### 4.17 Immunofluorescence staining

Frozen OCT blocks of tumors and LNs were cut using a cryostat (Leica) into 8‐μm thick sections and cells were incubated in glass‐bottomed well plate (Lab Tek™). Meanwhile, cell samples were also stained by the following procedure. The samples were stained using anti‐pan‐cytokeratin (AE1/AE3, sc‐81,714, SCBT), anti‐Melan‐A (ab210546, Abcam), anti‐LYVE‐1 (ab14917, Abcam), anti‐fibronectin (NBP1‐91258, Novus Biologicals), anti‐CD31 (14–0311‐82, Invitrogen), anti‐caspase‐3 (4‐1‐18, BioLegend), anti‐mouse/human Ki‐67 (11F6, BioLegend) antibodies, or anti‐HSP70 (HPA052504, Sigma‐Aldrich). Dye‐conjugated secondary antibodies (711‐585‐152, 711‐545‐152, 712‐545‐150, 712‐585‐153, Jackson ImmunoResearch) were used for the addition of the fluorescence signals. DAPI (VECTASHIELD, Vector Laboratories Burlingame, CA) was used to stain the cell nuclei. The stained tissue sections were imaged using a fluorescent confocal microscope and an EVOS FL2 auto microscope or FluoView FV‐10i Olympus Laser Point Scanning Confocal Microscope (Olympus, Center Valley, PA). After splitting each color channel, the fluorescent area for each color channel was measured with ImageJ (National Institutes of Health; Bethesda, MD). The expression level for each marker was calculated on the basis of normalization with the DAPI signal.

### 4.18 Hematoxylin and eosin staining

Major organs were immediately fixed in 4% paraformaldehyde, embedded in paraffin, and sliced into 5‐μm‐thick sections, which were then stained with H&E for histological examination. Organ histology was viewed and imaged under light microscopy (EVOS FL2).

### 4.19 Serological assessment of kidney damage in CLC‐injected mice

BUN of the C57BL/6 mice serum following *iv* injection of CLCs (two times per week for 2 weeks, total dose of CLC; 1.06 mg/kg) was measured by the Infinity Urea kit (Thermo Fisher Scientific) and compared against a standard BUN solution of 100 mg/dl (Sigma‐Aldrich) using a VersaMax microplate reader (Molecular Devices Corp., Sunnyvale, CA), as per the protocol provided by Thermo Fisher Scientific. The creatinine (Cr) of the serum following *iv* injection of CLCs (two times per week for 2 weeks, total dose of CLC; 1.06 mg/kg) was measured using the microplate reader, according to the instructions provided in the Mouse Creatinine Assay Kit (Crystal Chem, USA).

### 4.20 Statistical analysis

All statistical analysis was conducted using GraphPad Prism 7 software (GraphPad Software, Inc., CA). The expression level of each marker in the immunostaining experiments was determined by dividing each DAPI level. The sample size (*n*) in the experiments was as follows: six for tumor growth analysis, six for lung metastatic foci assay, three for the fluorescent biodistribution, and three for the tissue immunostaining study. The fluorescence in vitro assay was independently conducted three times for statistical analysis. All data are expressed as the mean ± SD from at least three independent samples or experiments. Differences between the two groups were analyzed by an unpaired Student's t‐test. Comparisons between multiple groups were determined using one or two‐way analysis of variance with Holm‐Sidak's post hoc test. A *p* value <0.05 was considered statistically significant (*p < 0.05, **p < 0.01, ***p < 0.001, and ****p < 0.0001).

## 5 CONCLUSION

In summary, CLC has significant potential to replace the antibodies used currently as delivery agents in ADCs, as it circumvents the complicated process of antibody optimization^6^ as well as the limited targeting efficacy^6^, ^7^ of immunogenic and large‐sized antibodies. Therefore, we expect CLC‐drug conjugates to open a new window for targeted, protein‐based delivery of drugs in cancer, as it may target metastatic lesions to enhance the survival rates in these cancer patients.

## AUTHOR CONTRIBUTIONS

**Sungwook Jung:** Conceptualization (lead); data curation (lead); formal analysis (lead); investigation (lead); methodology (lead); validation (lead); visualization (lead). **Liwei Jiang:** Conceptualization (equal); data curation (lead); formal analysis (lead); investigation (equal); methodology (equal). **Jing Zhao:** Data curation (supporting); investigation (equal); methodology (equal). **Leonard D. Shultz:** Funding acquisition (equal); resources (equal); supervision (equal). **Dale L. Greiner:** Methodology (equal); resources (equal); supervision (equal). **Munhyung Bae:** Data curation (supporting); methodology (supporting). **Xiaofei Li:** Data curation (supporting); methodology (supporting). **Farideh Ordikhani:** Conceptualization (supporting); formal analysis (supporting); methodology (supporting). **Rui Kuai:** Supervision (supporting); validation (supporting). **John Joseph:** Data curation (supporting); formal analysis (supporting); methodology (supporting). **Vivek Kasinath:** Data curation (supporting); formal analysis (supporting); funding acquisition (equal); supervision (supporting); validation (supporting). **David R. Elmaleh:** Conceptualization (equal); formal analysis (supporting); methodology (supporting); resources (supporting); supervision (supporting). **Reza Abdi:** Conceptualization (lead); data curation (lead); formal analysis (lead); funding acquisition (lead); investigation (lead); methodology (lead); project administration (lead); resources (lead); software (lead); supervision (lead); validation (lead); visualization (lead).

## CONFLICT OF INTEREST

All authors declare no conflict of interests.

### PEER REVIEW

The peer review history for this article is available at https://publons.com/publon/10.1002/btm2.10273.

## Supporting information


**Appendix S1**: Supporting information

## Data Availability

All data generated or analyzed for this study are available from the corresponding author upon reasonable request.
