# Supplementary material for: Clathrin light chain‐conjugated drug delivery for cancer
Source: Bioeng Transl Med. 2022 Nov 28;8(1):e10273. doi: 10.1002/btm2.10273 (PMC9842032; doi:10.1002/btm2.10273)
Supplement: Supplementary file 1 — Appendix S1: Supporting information [file BTM2-8-e10273-s001.docx]

Supporting Information

**Clathrin light chain-conjugated drug delivery for cancer**

Sungwook Jung^1,†^, Liwei Jiang^1,†^, Jing Zhao^1^, Leonard D. Shultz^2^, Dale L. Greiner^3^, Munhyung Bae^4^, Xiaofei Li^1^, Farideh Ordikhani^1^, Rui Kuai^5^, John Joseph^5^, Vivek Kasinath^1^, David R. Elmaleh^6^, and Reza Abdi^1,*^

^1^Transplantation Research Center and Renal Division, Brigham and Women’s Hospital, Harvard Medical School, Boston, MA 02115, USA

^2^Department of Immunology, The Jackson Laboratory, Bar Harbor, ME 04609, USA

^3^Department of Molecular Medicine, University of Massachusetts Medical School, Worcester, MA 01605, USA

^4^Department of Biological Chemistry and Molecular Pharmacology, Harvard Medical School, Boston, MA 02115, USA

^5^Center for Nanomedicine and Division of Engineering in Medicine, Department of Medicine, Brigham and Women’s Hospital, Harvard Medical School, Boston, MA 02115, USA

^6^Department Department of Radiology, Massachusetts General Hospital, Harvard Medical School, Boston, MA 02114, USA

^†^These authors contributed equally to this work

*Corresponding author. Email: [rabdi@rics.bwh.harvard.edu](mailto:rabdi@rics.bwh.harvard.edu)


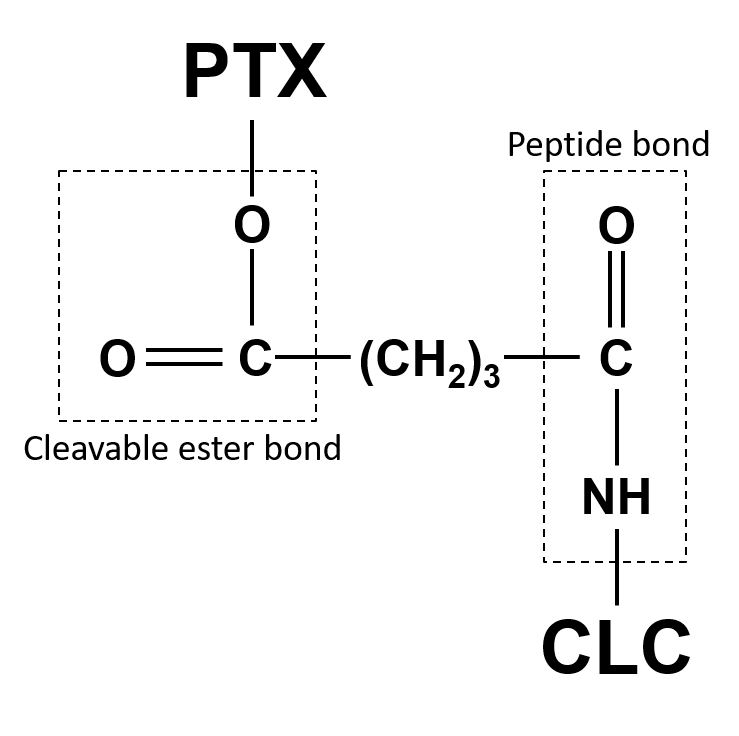


**Figure S1.** The chemical structure of linker in clathrin light chain-paclitaxel (CLC-PTX) conjugate.


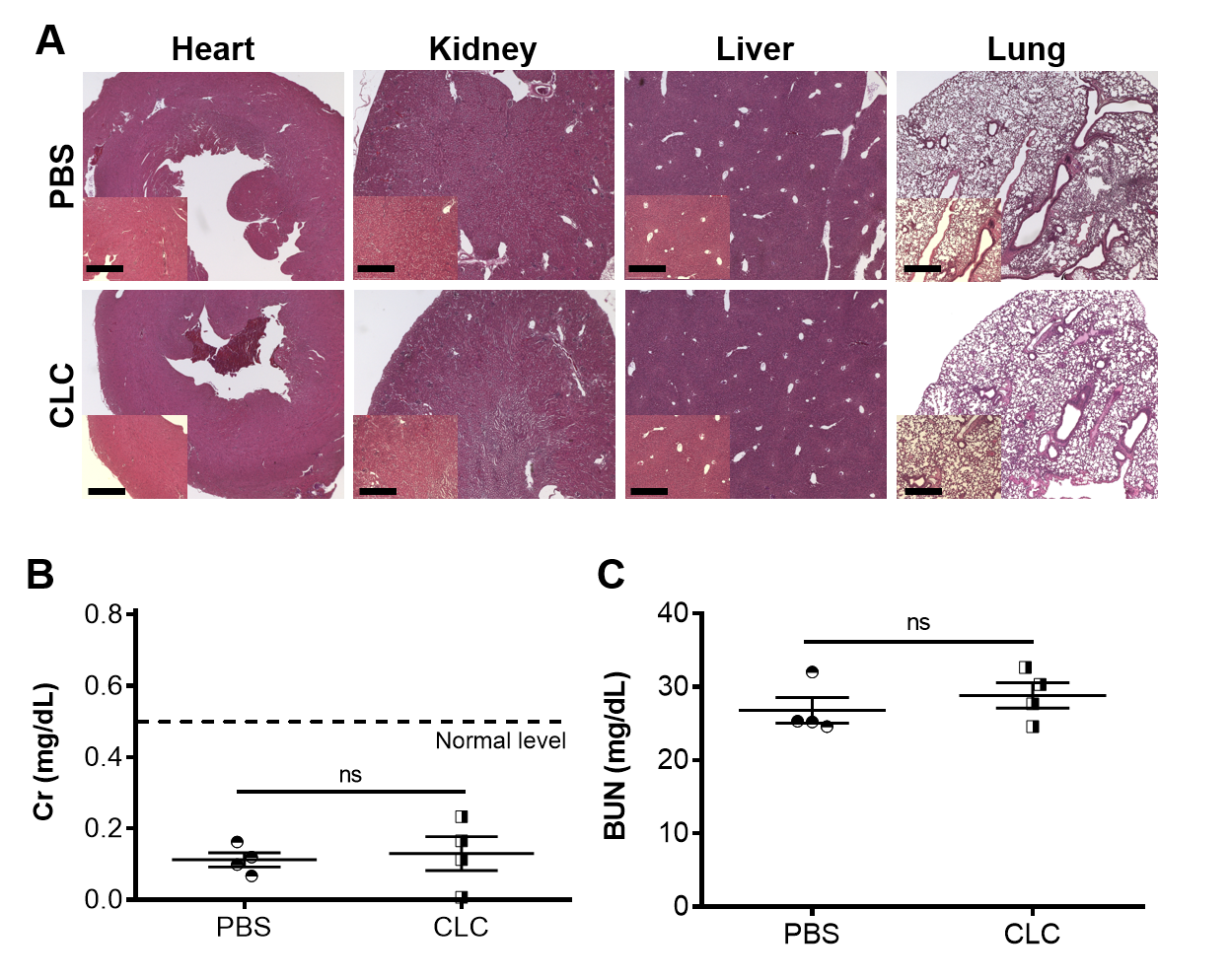


**Figure S2.** Toxicity assay of CLC. (A) Light micrographs of H&E-stained sections of heart, kidney, liver, and lung in C57BL/6 mice after treatment of PBS or CLC for two times per week for 2 weeks (total dose of CLC; 1.06 mg/kg). Scale bar: 100 μm. n = 3. (B–C) Serum creatinine (B) and BUN (C) of C57BL/6 mice after treatment with PBS or CLC two times per week for 2 weeks (total dose of CLC; 1.06 mg/kg). The data are represented by means ± SD (n = 3, ^ns^*p* > 0.05). The significance was determined by Student’s t-test.


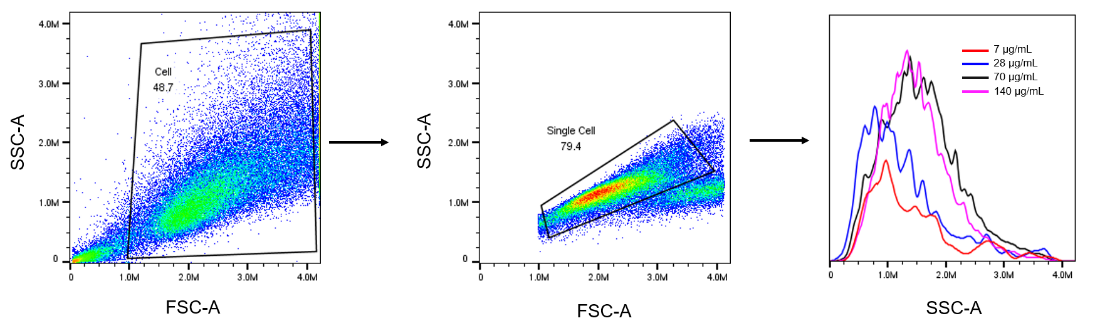


**Figure S3.** Flow cytometric analysis of 4T1 cells following incubation with different concentrations of CLC-Alexa 594.


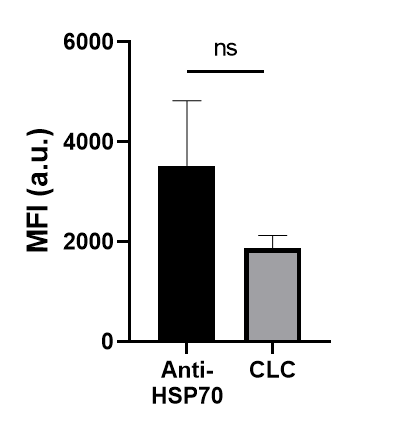


**Figure S4.** Fluorescence signal in flow cytometric analysis of 4T1 cells following incubation with either anti-HSP70-Alexa 594 or CLC-Alexa 594 (Student’s t-test).


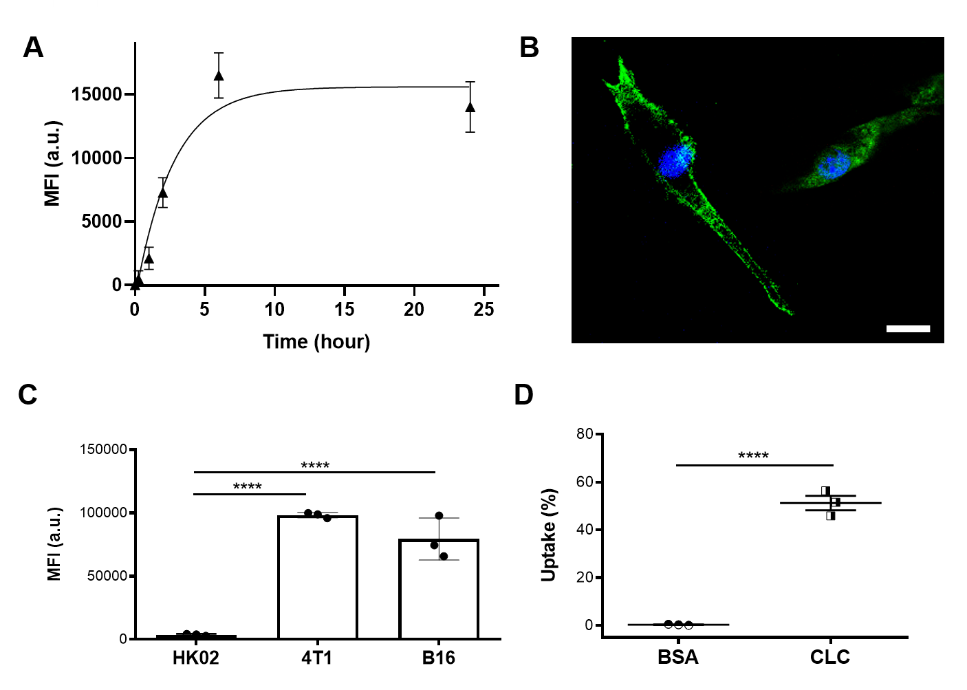


**Figure S5.** (A) Fluorescence signal of CLC-Alexa 594 (10 μg/mL) following incubation in 4T1 cells over time. (B) Confocal fluorescence micrograph of B16 cells stained for HSP70 (green). Blue: DAPI, Scale bar: 25 μm. (C) Expression level of HSP70 in HK02, 4T1, and B16 cells. One-way ANOVA with Holm-Sidak’s post-hoc (*****p* < 0.0001). (D) Uptake *in vitro* of CLC or BSA by 4T1 cells (2 h, 10 μg/mL). *****p* < 0.0001, Student t-test test.

**Figure S6.** Fluorescence intensity from livers, kidneys, and tumors of 4T1 tumor-bearing mice after injection of CLC-PTX*. Data were analyzed by one-way ANOVA with Holm-Sidak’s post-hoc (^ns^*p* > 0.05, ***p* < 0.01, ****p* < 0.001). The data are represented by means ± SD.


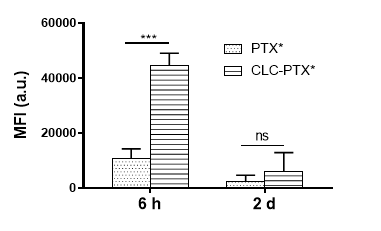


**Figure S7.** Fluorescence signal of PTX* or CLC-PTX* in serum 6 h and 2 d after injection in BALB/c mice. Student t-test (^ns^*p* > 0.05, ****p* < 0.001).

Table S1. A list of proteins with the highest docking scores to CLC.

| **Accession #** | **Name** | **Docking score** |
| --- | --- | --- |
| sp\|P61204\|ARF3 | ADP-ribosylation factor 3 OX=9606 GN=ARF3 PE=1 SV=2 | 13 |
| sp\|P34931\|HS71L | Heat shock 70 kDa protein 1-like OX=9606 GN=HSPA1L PE=1 SV=2 | 10 |
| sp\|P62140\|PP1B | Serine/threonine-protein phosphatase PP1-beta catalytic subunit OX=9606 GN=PPP1CB PE=1 SV=3 | 7 |
